# Autophagy in bone homeostasis and the onset of osteoporosis

**DOI:** 10.1038/s41413-019-0058-7

**Published:** 2019-10-03

**Authors:** Xing Yin, Chenchen Zhou, Jingtao Li, Renkai Liu, Bing Shi, Quan Yuan, Shujuan Zou

**Affiliations:** State Key Laboratory of Oral Diseases, National Clinical Research Center for Oral Diseases, West China Hospital of Stomatology, Sichuan University, Chengdu, 610041 China

**Keywords:** Osteoporosis, Bone

## Abstract

Autophagy is an evolutionarily conserved intracellular process, in which domestic cellular components are selectively digested for the recycling of nutrients and energy. This process is indispensable for cell homeostasis maintenance and stress responses. Both genetic and functional studies have demonstrated that multiple proteins involved in autophagic activities are critical to the survival, differentiation, and functioning of bone cells, including osteoblasts, osteocytes, and osteoclasts. Dysregulation at the level of autophagic activity consequently disturbs the balance between bone formation and bone resorption and mediates the onset and progression of multiple bone diseases, including osteoporosis. This review aims to introduce the topic of autophagy, summarize the understanding of its relevance in bone physiology, and discuss its role in the onset of osteoporosis and therapeutic potential.

## Introduction

Life forms have a dynamic nature. Within organisms, energy is constantly produced and consumed, and chemicals, including protein, fat, and sugar, undergo constant synthesis and degradation. A stable chemical and energy intake, either in the form of food absorbed in the digestive system or sunshine converted in the leaves, is indispensable for life to survive and multiply. For a long time, people believed that energy or chemicals from the outside environment are the sole source to support synthesis in vivo and maintain homeostasis. Later, we recognized that, under adverse environments, such as starvation, intracellular recycling of chemicals, including protein, fat, and minerals, became a backup solution to maintain the minimal amount of synthesis and energy production needed for survival.^1^ Furthermore, it has now been acknowledged that even under normal physiological conditions, the substrates for most intracellular synthetic processes in our body are mainly derived from the degradation, reformation, and reuse of contents that are already present. Such recycling characteristics become another intrinsic definition of life and are mainly achieved through a biological process named autophagy.^2^

Generally, autophagy is a highly conserved intracellular catabolic process during evolution, in which cytoplasmic components are degraded for nutrient and/or energy generation. At the very beginning, the physiological function of autophagy was limitedly recognized as merely a way of transporting intracellular components to the lysosome. Ever since the identification of autophagy-related genes and the molecules involved in membrane dynamics during autophagy, however, significant progress has occurred regarding the broad participation of autophagy in almost all biological processes. Autophagy has been identified as a critical player in both physiological processes and the onset and progression of numerous pathological conditions related to metabolic dysregulation,^3^ including cancer,^4^ neurodegenerative disorders,^5^ aging,^6^ and bone-related diseases.^7^ Under physiological conditions, autophagy is responsible for the removal of damaged or excessive organelles, whereas under pathological conditions, autophagy helps in the redistribution of intracellular nutrients to meet the substance and energy requirement for survival. Autophagy controls the energy and chemical homeostasis of each single cell and various tissue types, including bone.^8^

In mammals, bone assumes multiple important functions, providing protection to vital organs, attachments for skeletal muscles, niche sites for blood cell synthesis, a form of storage for mineral ions, and secretion organs of hormones.^9^ To fulfill the above-mentioned roles, our skeletal system undergoes a constant remodeling cycle.^10^ MSC (mesenchymal stem cell)-derived osteoblasts lining the surface of bone synthesize and secrete bony matrix.^11^ The matrix-embedded osteoblasts further differentiate into long-lasting osteocytes, and the latter forms a mechanosensing network within the bone.^12^ At the same time, multinucleated osteoclasts derived from hematopoietic stem cells constantly degrade and resorb their surrounding bone matrix. Normally, a dynamic balance between the formation and degradation of the bone is constantly coordinated.^13^ In this way, the mass, structure, and functions of bone tissue are unsurprisingly sensitive to either intrinsic or extrinsic stimuli.

When the equilibrium between bone formation and bone degradation is disturbed, pathological conditions occur. Excessive bone-formation activity leads to overmineralization and excessive mass of bone, which is called osteopetrosis, also known as marble bone disease.^14^ However, when an imbalance toward bone degradation predominates, increased bone loss leads to reduced mass and undermined structure of bone, which is frequently called osteoporosis.^15^ Osteoporosis is a systemic bone degenerative disease characterized by progressive loss of bone mass and significant degradation of bone mechanical properties, which subsequently leads to bone fragility and susceptibility to fractures. This phenomenon is usually correlated with the progress of aging and significantly worsens the quality of life and longevity of the elderly population.^16,17^

Considering the “recycling” property of autophagy and the dynamic synthesis and degradation processes within bone, it is not surprising to find that autophagy is highly involved in the metabolism of bony tissue. All three types of bone cells, osteoblasts, osteocytes, and osteoclasts, demonstrate a basal level of autophagic activity. Multiple components of the autophagic pathway contribute to mediating the survival and functioning of osteoblasts, osteocytes, and osteoclasts.^18–20^ Increasing evidence suggests that an appropriate level of autophagy enables osteoblasts, osteocytes, and osteoclasts to survive hypoxic, nutrition-deficient, or even hypertonic environments. In addition to survival, the level of autophagic activities is associated with pre-osteoblast differentiation, osteoblast-osteocyte transition, and the genesis and functioning of osteoclasts.^18,21,22^

In addition to the correlation between autophagy and bone physiology, recent evidence suggests that autophagy plays a fundamental role in the onset and progression of pathological osteoporosis.^7,22,23^ The relationship between the autophagy pathway and osteoporosis was highlighted in a genome-wide association study of human wrist bone mineral density, where significant correlations between multiple autophagy-regulatory genes and bone mineral density were identified.^24^ In addition, selective modulation of autophagy-related genes in bone cells is sufficient to recapitulate the osteoporotic state in animal models.^18,25^ At the same time, modulation of autophagic activities has potential therapeutic value for the prevention and treatment of osteoporosis.^26,27^

This review summarizes the up-to-date research findings about the autophagic process and its role in skeletal homeostasis and the onset of osteoporosis, and discusses both the potential and challenges in the therapeutic application of autophagy modulators.

## Autophagy: self-eating for survival

Autophagy, which originates from the Greek roots autos- (self) and phagein- (eat), is a lysosomal pathway responsible for the recycling of unnecessary cell organs and excessive nutrients and the elimination of metabolic wastes and intracellular pathogens.^26–28^ Such an intracellular “self-eating” process plays a critical role in maintaining the survival of multiple cell lineages.^29^

**Fig. 1:**
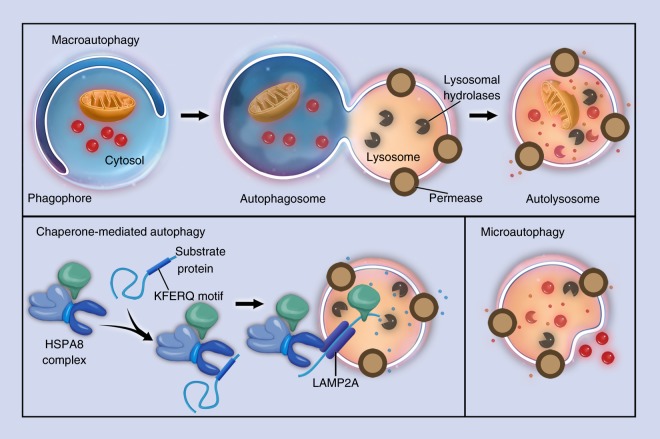
Three types of autophagy. Schematic illustrations of (**a**) macroautophagy, (**b**) chaperone-mediated autophagy, and (**c**) microautophagy

In mammals, three types of autophagy with distinct morphological features and different regulatory mechanisms have been described: chaperone-mediated autophagy, microautophagy, and macroautophagy. In chaperone-mediated autophagy, cytoplasmic proteins are not sequestered and are delivered to the lysosome by chaperone proteins rather than membranous structures.^30^ Chaperones match with proteins containing a specific pentapeptide motif, and then, these substrates are unfolded and translocated individually and directly across the membrane of lysosomes.^31^ In microautophagy, the lysosome directly captures a small amount of nearby cytoplasm by forming invaginations or protrusions on its membrane, requiring little assistance from organelles outside the lysosome.^32–34^ In macroautophagy, the capture and delivery of intracellular substances is symbolized by the formation of autophagosomes. The autophagosomes are constituted by newly formed bilayer membranes, and can enclose damaged organelles, intracellular pathogens, and protein aggregates to achieve the sequestration process.^35^ Autophagosomes are then incorporated by lysosomes to finish the delivery and digest the contents (Fig. 1). Compared with microautophagy, autophagosomes can capture cytoplasm far away from the lysosome. Macroautophagy is regulated by a group of evolutionarily conserved genes named *Atg* (autophagy-related genes). The *Atg* genes have diverse functions, including the transportation of both intracellular and extracellular cargos and coordination of intracellular communication with all kinds of signaling pathways. The *Atgs* include approximately 20 members. During the initiation and maturation of autophagosomes, *Atgs* are actively involved in the formation of double-membrane vesicles and the delivery of cargos in autophagosomes to lysosomes.^36^ Meanwhile, *Atgs* may interact with signaling pathways other than autophagic ones. For example, *Atg7* is downstream of FGF signaling in the regulation of endochondral bone formation and long bone growth.^37^

Among the three types of autophagy, macroautophagy has the strongest connection with cell biology, physiology, and disease, and will hereinafter be referred to as “autophagy” in this review.

### A highly organized degradation program

**Fig. 2:**
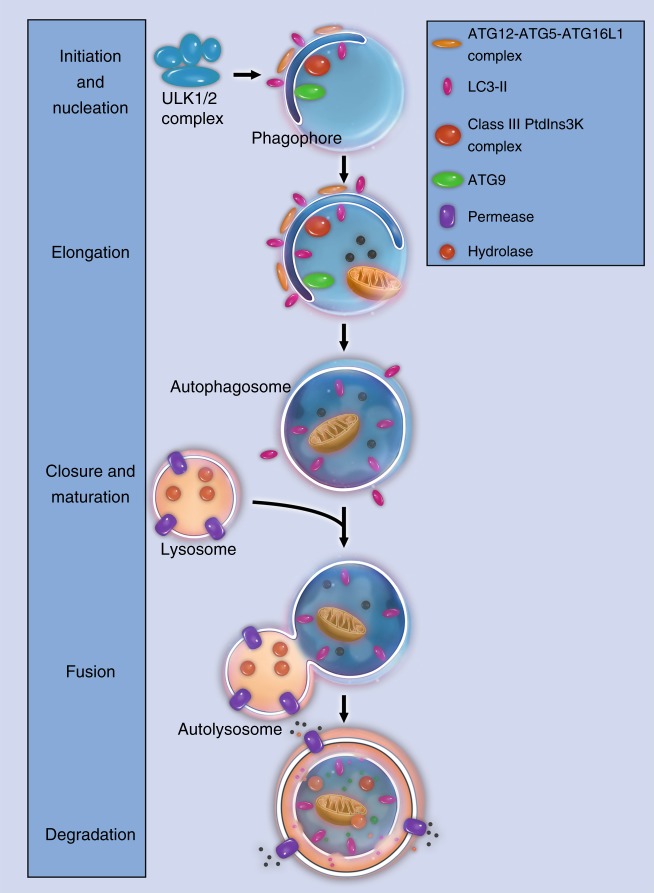
Major stages in the autophagic process. Schematic illustrations of major stages in the autophagic process: initiation and nucleation, elongation, closure and maturation, fusion and degradation

Autophagy is a highly conserved cellular process during evolution.^2^ From yeast to vertebrates, autophagy works in concert with the UPS (ubiquitin–proteasome system) to maintain cellular homeostasis.^38^ Closer examination defines the autophagic process into four major stages: initiation/nucleation, elongation, degradation, and termination (Fig. 2).^32,35^

Autophagy starts with activation of the ULK1 complex, which is composed of ULK1, ATG13, ATG101, and FIP200. The ULK1 complex originally associates with the mammalian target of rapamycin complex 1 (mTORC1) complex. At the initiation of autophagy, ULK1 is dephosphorylated, and the ULK1 complex dissociates from mTORC1.^39^ The activated ULK1 complex recruits another multiprotein complex, known as the class III phosphatidylinositol 3-kinase (PI3K) complex, to the site of autophagy initiation. The PI3K complex is composed of beclin-1, Vps15, Vps34, Ambra1, UVRAG, and more.^28,40^ Ambra1 interacts with TRAF6 and leads to self-association and stabilization of these complexes. In this process, a membrane fragment usually known as a phagophore is formed.^41^

In the next step, ATG proteins participate in the elongation of the phagophore. The ATG proteins aggregate and form a ubiquitin-like conjugation system, ATG12–ATG5–ATG16L, which facilitates the assembly of LC3 (microtubule-associated protein 1A/1B-light chain 3) with PE (phospholipid phosphatidylethanolamine). LC3-PE, which is also called LC3-II, then incorporates into the phagophore membrane and contributes to the elongation and closure of the autophagosome.^32,42^

Autophagosomes mature by fusion with intracellular endocytic components, including endosomes and lysosomes,^43^ turning the environment inside the autophagosome acid. Proteins involved in vesicular transport, such as dynein, and membrane fusion, including Rab7, SNARES, and ESCRT, facilitate the maturation of autophagosomes.^44^ Some proteins on the surface of autophagosomes, including p62, optineurin, NDP52, NBR1, and Alfy,^45,46^, are responsible for the sequestration of degradation targets. During the degradation stage, entrapped intracellular macromolecules are broken down into amino acids, lipids, nucleotides, and energy for the purpose of future intra- and extracellular processes.^47^

Termination of autophagy is achieved through a negative feedback mechanism. Nutrients produced in autophagosomes reactivate the mTOR (mammalian target of rapamycin) pathway, and the latter generates proto-lysosomal tubules or vesicles. These tubules and vesicles extrude from the autolysosomes and eventually mature into lysosomes again. Such a termination process serves as the closing stage of the autophagic machinery and has been validated in various species.^48,49^

Critical molecules in the above-described autophagic process have been employed for the assessment of autophagy flow. For example, Beclin-1 is fundamental for the formation of PI3K complexes and, therefore, has been commonly used as a marker of autophagic initiation.^48^ LC3-II found within the autophagosome membrane has been widely used as a specific autophagosome marker.^32,49^ Analyses of the combined expression of proteins p62 and LC3-II are commonly used to assess autophagic flow.^50,51^

In addition to degrading intracellular contents, autophagy can target extracellular cargo. Several core ATG proteins are involved in the phagocytosis of unwanted extracellular components. During such ATG-assisted phagocytosis, extracellular targets, such as pathogens and apoptotic cells, are engulfed by single-layered vacuoles and then labeled by LC3, which delivers the contents to lysosomes for degradation.^52,53^

### A target-specific digestion process

**Fig. 3:**
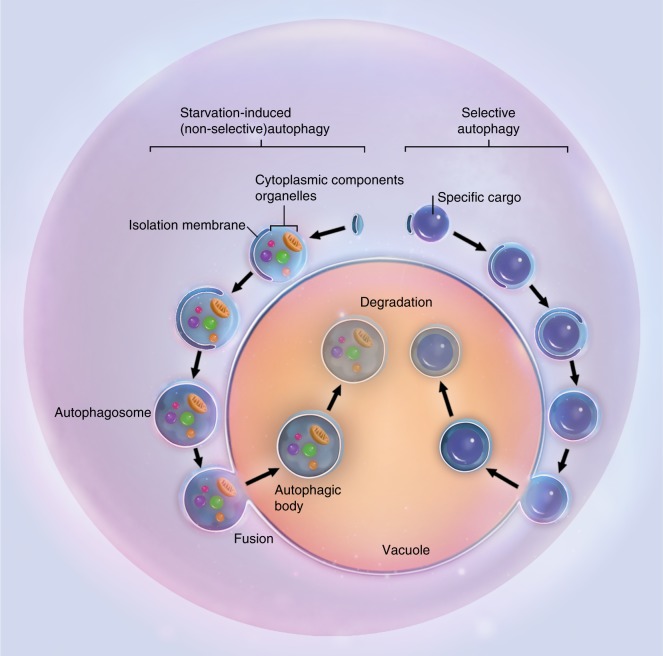
Non-selective and selective autophagy. Schematic illustrations of starvation-induced nonselective autophagy and target-specific selective autophagy

For a long time, autophagy has been recognized as being nonselective for its degradation substrates.^50,51^ The simultaneous observation of multiple intracellular components in double-membrane vesicles has been employed as a standard for the identification of autophagy. While this is often true when autophagy is induced in stressed conditions such as starvation, recent evidence suggests that autophagy required during the maintenance of cell homeostasis could be highly specific.^51,54–57^ Actually, autophagy can be extremely specific in choosing the cargo for autophagosomes. An intricate system is in charge of barcoding and selectively sequestering the substrates for autophagy. This process is termed selective autophagy and is more critical during diseases. Substrates commonly sequestered and digested during selective autophagy may include ubiquitinated proteins, peroxisomes, and mitochondria (Fig. 3).^58–60^

The best demonstration of protein-specific autophagic degradation is the ubiquitin-binding protein SQSTM1 (sequestosome 1), also called p62, on the autophagosome surface. P62 can capture ubiquitinated proteins and binds to the membrane component LC3-II.^61^ While delivering the target substrates to the inside of the autophagosome, p62 itself is also internalized and degraded. P62 is considered one of the major digestive substrates for autophagosomes, and therefore, increased expression of p62 usually indicates a decline in the autophagic process. NBR1 autophagy cargo receptor and OPTN (optineurin) are other receptors that specifically deliver ubiquitinated proteins or pathogens to autophagosomes.^62–64^ Ubiquitination-involved autophagy is most active in the clearance of bacteria. When the pathogens are specifically engulfed and digested during autophagy, the process is named xenophagy.^62,65^

When peroxisomes are selectively degraded during autophagy, the process is termed pexophagy. LC3-II on the surface of autophagosomes could specifically bind to peroxisomal biogenesis factor 14 (PEX14), a component of the peroxisomal translocon complex. In this way, peroxisomes are specifically recognized by autophagosomes during starvation.^66^ Actually, in certain organs, autophagy could accomplish up to 70%–80% of peroxisome turnover under normal growth conditions.^67^ Considering the widespread influence of peroxisomes on metabolism and the negative consequences of peroxisomal dysfunction in health, specific pexophagy must be a critical player in maintaining organism homeostasis.^58^

Another selective target for autophagy is the mitochondria, and the specific autophagic degradation process is termed mitophagy. For intact mitochondria, LC3 and gamma-aminobutyric acid receptor-associated protein (GABARAP) on expanding phagophores could specifically bind to a complex on the outer membrane of the mitochondria called BNIP3L/NIX and subsequently turned the following maturation and degradation stages into mitophagy.^68^ For damaged mitochondria, this selective capture process is slightly altered. When a mitochondrion loses its integrity, the kinase PINK1 accumulates on its outer membrane and specifically binds to the cytosolic E3 ubiquitin ligase PARK2/Parkin. The latter then ubiquitinates mitochondrial substrates and initiates mitophagy.^68^ Mitophagy is responsible for the routine turnover of mitochondria under normal conditions^69^ and is also important to the differentiation of certain cell lineages.^70–72^ For instance, the maturation of mammalian red blood cells requires the elimination of the mitochondria from immature cells, and this process is mainly achieved through mitophagy.^72–74^ Insufficient clearance of the damaged mitochondria has been associated with multiple pathological conditions. In autosomal recessive Parkinson’s disease, the genes encoding PINK1 and PARK2 are mutated,^75,76^ suggesting the critical role of mitophagy in maintaining cellular and organismal health.

In addition, autophagy could selectively target misfolded proteins that tend to aggregate. These inappropriate protein products are usually involved in pathological states, including neurodegenerative, skeletal, cardiac muscle, and liver diseases.

Selective autophagy demonstrates concurrent mechanisms in recognizing specific digestive substrates. These coexisting mechanisms cooperate with each other in a redundant manner to ensure efficient digestion of the unwanted material.

### Physiological and pathological autophagy inducers

Autophagy is initiated with either physiological signals or pathological stimuli. At the physiological basal level, the autophagic process is constitutive at a low level in all cells, serving as a quality control mechanism to remove flawed organelles and proteins.^77^ The basal level of autophagic activity varies among different cell lineages and tissue types.^78^ Generally, such basal-level autophagy is more critical for highly or terminally differentiated, long-lived cells, such as neurons, myocytes, and osteocytes, in maintaining their homeostasis and functioning.^79^

A wide range of extracellular and intracellular stresses, including nutrient or energy starvation, hypoxia, disturbance in growth factor level, or pathogen invasion, induce an increased rate of autophagy to recycle cytoplasmic components into metabolites and biosynthetic processes or to eliminate pathogens, allowing for cell survival.^80–82^

The cAMP-dependent PKA (protein kinase A) pathway and mTOR pathway are involved in the initiation of autophagy induced by nutrient starvation. These two pathways sense levels of carbon and nitrogen.^83^ In conditions where nutrients are sufficient, PKA inhibits autophagy by inducing phosphorylation of LC3.^84,85^ A high level of amino acids upregulates RAG (RAS-related small GTPases), which subsequently activates MTORC1 and inhibits autophagy.^86,87^ While the PKA pathway and TOR pathway could both independently affect the Atg1/Atg13 protein kinase complex and its subsequent autophagic process,^88^ there might also be crosstalk between them. For example, the PKA and TOR pathways are both nutrient-sensitive, and their function overlaps in regulating cell proliferation.^89^ The regulation of ribosome formation from TOR is achieved partially via PKA signaling.^90^ Thus, these two pathways might coordinate in the fine-tuning of the level of autophagic activity.

In mammals, PKA can phosphorylate and activate MTORC1.^91,92^ PKA can also indirectly activate the MTORC1 complex through the inactivation of AMP-activated protein kinase (AMPK).^93^ In addition to being a substrate for PKA, AMPK is a major intracellular energy-sensing kinase. AMPK senses AMP/ATP levels and regulates a wide variety of cellular processes, including autophagy.^94,95^ At a low energy level, AMP binding activates the kinase activity of AMPK, and the latter phosphorylates and activates the TSC1/2 complex, which indirectly inhibits the function of MTORC1.^96,97^ At the same time, AMPK may directly downregulate the activity of MTORC1.^98,99^ Evidence also suggests that AMPK can phosphorylate and activate ULK1, which subsequently induces autophagy.^100–103^

Hypoxia and disturbance in growth factors also induce autophagy via the TOR pathway. Even with adequate nutrients and energy supply, hypoxia or growth factor fluctuation can inhibit MTOR1 and result in the induction of autophagy.^95,104,105^

In the case of endoplasmic reticulum (ER) stress, the increase in cytosolic Ca^2+^ concentration activates calcium/calmodulin-dependent protein kinase kinase 2/beta (CAMKK2/CaMKKb), and the latter subsequently activates AMPK and induces autophagy.^106^ At the same time, when unfolded proteins accumulate in the ER under stress conditions, unfolded protein response (UPR) signaling is triggered to induce autophagy. The ending point of ER stress-induced autophagy is still unclear. Both enhanced survival and increased autophagic apoptosis have been reported in the current literature.^82,107^

In addition to trafficking substrates to autophagosomes for degradation, ATG proteins are involved in the process of exocytosis. LC3 regulates this autophagic-independent exocytosis, which expels pathogens that either locate inside the autophagosomes or are labeled by LC3.^52^ In the case of virus invasion, ATG proteins are involved in the formation of membrane-associated replication units of virus or bacteria, including hepatitis C and *Brucella abortus*.^108,109^

Generally, autophagy contributes to cell homeostasis by specifically eliminating flawed or redundant organelles and boosting chemicals and energy recycling.^98^ Under most conditions, autophagy serves as a cytoprotective mechanism, but could potentially turn deleterious if it becomes uncontrolled. Autophagic dysfunction is thus associated with a wide variety of human pathological conditions, including skeletal system disorders and diseases.^36,110^

## Autophagy regulates bone formation

**Fig. 4:**
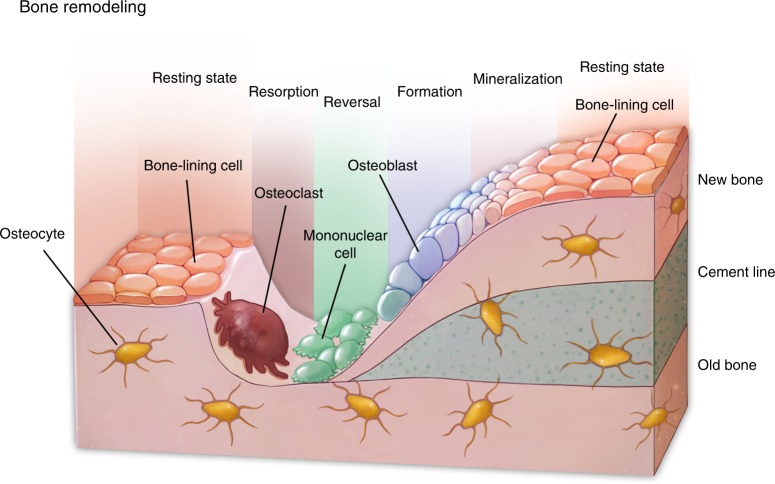
Bone remodeling and skeletal system homeostasis. Osteoblasts, osteocytes, and osteoclasts are tightly linked to regulate bone remodeling and orchestrate the homeostasis of the skeletal system

The development, growth, and maintenance of the skeletal system are in dynamic balances and are highly sensitive to factors, including mechanical stimulus and hormone fluctuation.^111^ Mesenchymal cells in the bone marrow or lining the periosteum include a multipotent population that is capable of osteogenic, adipogenic, and chondrogenic differentiation. Mesenchymal-derived osteoblasts are responsible for the synthesis, secretion, and mineralization of the bony matrix and further differentiate into osteocytes when embedded in the mineralized matrix.^112^ Osteocytes are embedded in the bone matrix and form a cellular network that regulates skeletal remodeling.^113^ Hematopoietic stem cell-derived multinucleated osteoclasts execute bone resorption during remodeling. Osteoclasts secrete degradative enzymes to the bony surface to dissolve the minerals and digest the bony matrix and recycle the degraded contents via endocytosis.^114^ These three types of cells are tightly linked to orchestrate the homeostasis of the skeletal system (Fig. 4). For example, the physiological stimulus for osteoblast-mediated new bone formation is strongly connected with the resorptive activities of osteoclasts during which growth factors such as TGF-β1 (transforming growth factor β1),^115^ IGFs (insulin-like growth factors),^116^ and bone morphogenetic proteins (BMPs) were released from the extracellular matrix of the bone.^112,117,118^ Autophagy is actively involved in each component of this cross-talking network.

Phenotypically, human genome­wide screenings have illuminated the correlation between single-­nucleotide polymorphisms of autophagy-­related genes and status.^119^ The expression of autophagic pathway-regulatory genes demonstrated a direct influence on the variation in bone mineral density (BMD) in the distal portion of the radius.^24,120^

Among cells with a high secretion capacity, autophagy controls the spatial localization of signaling complexes critical to protein synthesis.^121^ Such intracellular spatial localization has been revealed in the activation of Wnt^122^ and NF-kB^123^ signaling via autophagic degradation of specific pathway components. In addition to protein trafficking, autophagy orchestrates diverse cellular pathways involved in cell viability, cell renewal, and innate immune response. Since these pathways are critical to the differentiation of osteoblasts and osteoclasts, the importance of this autophagy-regulated spatial localization in regulating the anabolic and catabolic functions of bone cells is apparent. Since autophagy is an indispensable cell activity during embryogenesis, whole-body deletion of autophagy-related genes in animal models almost invariably causes death at birth.^79^ As a result, various cell-specific conditional knockout models have been established to explore the specific role of autophagy in osteoblasts, osteocytes, and osteoclasts.^124^

### Autophagy fine-tunes osteoblast differentiation and functioning

Osteoblasts are the primary constructors of bone. These mesenchymal-derived cells deposit the bone matrix via constant synthesis and section activities. Both survival and physiological functioning are closely regulated by autophagy.

In the bone marrow, a bone-fat balance has been described in the differentiation of mesenchymal stem cells. Aging, estrogen deficiency, or a high-fat diet would push MSCs toward adipogenic differentiation and result in compromised bone density. Autophagy has been correlated with the stemness maintenance of mesenchymal progenitors.^125^ MSCs were shown to accumulate autophagosomes in the stem state and deliver them to lysosomes once differentiation is initiated, suggesting that autophagy-related metabolism is tightly associated with MSC differentiation.^126^ More specifically, the activation of autophagy has been correlated with the osteogenic differentiation of MSCs through AMPK signaling pathways.^127^

An appropriate autophagy level is a prerequisite for the maintenance of homeostasis and survival of osteoblasts. In vitro studies demonstrated a negative correlation between the level of autophagy and oxidative stress. Pharmacological downregulation of autophagy leads to increased oxidative stress in osteoblast-like cells, whereas upregulation of autophagy in these cells is correlated with reduced oxidative stress and decreased apoptosis.^128^ Knockdown of autophagy-essential genes increased the level of oxidative stress in osteoblasts.^18^ Additional data suggested that the damage caused by oxidative stress to osteoblasts could be relieved by early initiation of autophagy, which might be achieved through the ER stress pathway. Estrogen has demonstrated inhibitory effects on the serum deprivation-induced apoptosis of osteoblasts, and part of this protective effect is achieved by promoting autophagy via the ER-ERK-mTOR pathway.^129^ In addition, autophagy demonstrated protective effects on osteoblasts from various toxic stimuli. For example, a high level of autophagic flux reduced cell death of osteoblasts exposed to lead chloride,^130^ and upregulation of the level of autophagic flux aided in the survival of differentiated bone cells in a stressful environment.^131^ In addition to survival, autophagy is closely related to the differentiation and mineralization of osteoblasts. The most direct proof of the role of autophagy in mineralization is the identification of apatite crystals in autophagy vacuoles.^18^ Inhibition of autophagic flux also blocked the outward transportation of the minerals from osteoblasts. Gene knockout of *Beclin-1* or *Atg7* resulted in deficient mineralization in an in vitro setting.^18^ The in vivo role of autophagy in mineralization has also been reported. Targeted *Atg5* deletion in osteoblasts led to a 50% reduction in the trabecular bone mass.^132^ Osteoblast­specific deletion of *Fip200* (FAK family–interacting protein of 200 kDa) undermines the terminal differentiation of osteoblasts, inhibits bone formation, and consequently leads to an osteopenia phenotype.^133^ Moreover, osteoblast-specific conditional deletion of *Atg7* led to decreased bone formation by triggering ER stress, and relief of ER stress by systemic delivery of phenylbutyric acid could restore the bone-formation balance.^134^ When *Atg7* was deleted in the entire osteoblast lineage using *Osterix-Cre* transgenic mice, the number of both osteoblasts and osteoclasts decreased, and the osteocytes demonstrated decreased cellular projections and increased ER retention.^135^

Autophagy is also actively involved in signaling pathways that are of confirmed significance to osteogenesis. For example, insulin-like growth factor-I (IGF-I) stimulates the osteogenic differentiation of osteoblasts, the function of which is at least in part achieved via activation of AMPK and upregulation of autophagy.^136^

In addition, one of the pro-osteogenic cascades induced by bone morphogenetic protein-2 (BMP-2) involves the activation of the autophagy-related factor *Atg7*, which subsequently targets *Wnt16* to activate metalloproteinase-13 and eventually osteoblastic differentiation.^137^

In addition to osteoblasts, chondrocytes are another critical cell population responsible for skeletal growth. Except for the craniofacial region, long bones form and grow via endochondral bone formation, during which chondrocytes undergo hypertrophy and active matrix secretion.^138^ In vitro studies revealed that the differentiation and mineralization of chondrocytes are positively correlated with the level of autophagy activity.^139^ During the postnatal growth of mice, autophagy is turned on in the growth-plate chondrocytes, and its level is closely correlated with the secretion of type II collagen, which is the major component of cartilage matrix. When *Atg7* is deleted specifically in chondrocytes, the synthesized type II procollagen could not be transported out but was retained within the ER. Furthermore, autophagy in growth plate chondrocytes was suppressed in *Fgf18* and *Fgfr4* deletion mice, resulting in osteopenia. This phenotype could be rescued by pharmacological activation of autophagy. These data suggest that autophagy serves as an effector for FGF signaling during endochondrial bone formation.^37^

Multiple pathways or growth factors with evident bone-regulating capacities demonstrate crosstalk with autophagic activities. BMPs are recognized as strong osteogenic growth factors, and members of the protein family have been successfully applied in clinical settings. BMPs directly bind to receptors on the surface of osteoblasts and activate the bone-formation process through the intracellular SMAD signaling system. The paracrine function of the BMPs could be adjusted by the level of its extracellular antagonists, noggin, chordin, and sclerostin.^140^ The evidence from a study in pancreatitis cells suggested that BMPs may antagonize the dampening effect of noggin on microtubule-associated protein 1 light chain 3 (MAP1LC3)-II levels and subsequently increase the expression levels of *Beclin-1* and lysosomal-associated membrane protein 2 (*Lamp2*). In this way, BMP ligands might be involved in the regulation of autophagy levels.

β-catenin-dependent canonical Wnt signaling is another osteogenic pathway that has been associated with autophagy. Wnt signaling is critical to the commitment of stem cells to the osteoblast lineage and the subsequent osteoblast differentiation. Wnt ligand triggers DVL (dishevelled segment polarity protein) recruitment to the plasma membrane by binding to the Frizzled receptor. DVL promotes MAP1LC3-mediated autophagosome recruitment, ubiquitination, and degradation by binding to SQSTM1. In addition, under stress conditions, DVL becomes ubiquitylated and recognized by p62, and p62 in turn promotes DVL aggregation and degradation by LC3-mediated autophagy.^122^ In this way, the Wnt signaling pathway is negatively associated with autophagy. Moreover, since activation of the WNT-CTNNB1 signaling pathway is critical to the pathogenesis of osteoarthritis, activation of Wnt signaling might suppress autophagic activity and increase osteoblast or chondrocyte apoptosis.

At the same time, multiple autophagy-related proteins have been suggested to more directly influence the biology of osteoblasts. For example, knock-in mutation of the autophagy cargo receptor NBR1 resulted in enhanced osteoblast differentiation and bone formation.^141^ NBR can identify ubiquitinated proteins by its ubiquitin-like modifier activating enzyme domain and deliver them to autophagosomes by binding to MAP1LC3 by its LC3-interacting domain LIR.^142^ Aberration in these two domains of NBR1 leads to increased expression of SQSTM1 on the surface of autophagosomes and many other cytoprotective factors.

Two families of transcription factors with evident functions in autophagic activities are involved in the survival, differentiation, and function of osteoblasts. Members of the forkhead box O (FOXO) transcription factor family are profoundly involved in cell biology, including proliferation, hypertrophy, differentiation, DNA repair, cell cycle, energy recycling, and glucose metabolism.^143^ FOXO activation enhances the level of autophagy by directly binding to the promoter regions of autophagy-related genes. Specific FOXO deletion resulted in increased oxidative stress and apoptosis in osteoblasts, while FOXO3 overexpression prevented aging-related bone loss.^144^ Considering the role of autophagy in maintaining cell survival and the onset of aging-related physiological changes,^145^ it is natural to speculate that FOXO participates in the regulation of bone cell homeostasis via its induction effect on autophagy.

Activating transcription factor 4 (ATF4) from the cAMP-responsive element binding (CREB) protein family is also linked with both osteoblast function and autophagic activity. ATF4 is required in both the bone formation and terminal differentiation of osteoblasts. Mutation or disturbance in the level of ATF4 has been associated with two human genetic skeletal system diseases, Coffin–Lowry syndrome and neurofibromatosis type I. ATF4 overexpression in fibroblasts could induce osteoblast-specific gene expression, osteocalcin synthesis, and aberrant mineral deposition.^146^ At the same time, ATF4 protects cells from amino acid starvation by enhancing the intakes of amino acids into cells. Interestingly, ATF4 is deemed an enhancer of cell survival and viability by upregulating the transcription of several autophagy genes, including microtubule-associated proteins 1A/1B light chain 3B (*Map1lc3b*) and *Atg5*. In addition, an aberrant increase in bone mass caused by neurofibromin-1 could be rescued by restricted amino acid intake.^147^

### Autophagy takes part in the maintenance of osteocyte homeostasis

Autophagy is also essential when osteoblasts are incorporated into the bone matrix, thus terminally differentiating into osteocytes.^148^ Osteocytes are terminally differentiated cells embedded in the niches delimited by mineralized bone matrix. In contrast to the short cell lifespan of osteoblasts, osteocytes are very long-lived cells and are closer to neurons than other bone or cartilage cells in morphology. First, osteoblasts undergo a drastic transition in cellular morphology and composition to become osteocytes, which requires active recycling of organelles.^149^ Second, with limited blood perfusion in the mineralized matrix, osteocytes are more susceptible to hypoxia and high oxidative stress^150^ and thus require a tighter budget on nutrient preservation. As discussed above, both of these aspects require active autophagy. Thus, not surprisingly, MAP1LC3 distribution is evident in a considerable proportion of embedded osteocytes, proving a basal level of autophagic activities.^131^

Specifically, osteocytes depend on autophagy to survive multiple adverse factors, including high ROS and hypoxia. When *Atg7* was specifically knocked out in osteocytes using *Dmp1 (dentin matrix protein 1)-Cre* transgenic mice, oxidative stress increased as measured by ROS production and p66 phosphorylation. Moreover, this deletion could significantly lower the level of bone mass and bone remodeling, resulting in a phenotype that mimics the process of bone aging.^151^

Moreover, the present evidence suggests that osteocytes demonstrate greater autophagic activity than their progenitors. The expression level of LC3 in osteocytes is significantly higher than in osteoblasts.^131^ In addition, autophagy was correlated with the survival of osteocytes in a hypoxic environment. Pharmacological activation of autophagic activity reduced osteocyte apoptosis under high oxidative stress.^148^ In vivo-specific deletion of the *Atg7* gene in osteocytes caused a significant reduction in bone mass, which was simultaneously associated with reduced osteoclast and osteoblast numbers and a disturbance of the balance between bone resorption and bone formation.^151^ Thus, autophagy plays a vital role in the survival of osteocytes and the maintenance of bone mass and remodeling.

In addition, in an ex vivo environment, autophagy in pre-osteocyte-like cells could be upregulated by nutrient starvation, hypoxia, or calcium stress,^131^ indicating the involvement of autophagy in the survival of osteocytes under stressed conditions. In addition, glucocorticoid treatment could increase the level of autophagic activity by up to 30-fold and profoundly influence osteocyte function.^152^

The major physiological function of osteocytes is to act as the mechanosensing system of skeletal tissue. The dendrite-like processes extending from osteocytes form a network and convert mechanical stimulus on the bone into biological signals that subsequently regulate the remodeling process. Primary cilium is an organelle that plays crucial roles in a variety of cellular functions, including mechanosensation. Both primary cilium and autophagic activity have been tightly linked to various types of bone diseases, and their interaction has been suggested recently. Generally autophagic activity is suppressed in cells with shorter cilia. Conversely, when autophagy is downregulated either by exogenous pharmaceuticals or the intrinsic absence of autophagy-related proteins, ciliogenesis is enhanced and larger cilia length is observed. Further studies suggested that cilia and autophagic activity reciprocally regulate each other through the mTOR signaling pathway and the ubiquitin–proteasome system, and there might exist a negative feedback mechanism between autophagy and ciliogenesis.^153^

Aside from the mechanism directly mediated by primary cilium, mechanical stimulus may activate autophagy levels in MSCs, osteoblasts, and even chondrocytes. Mechanical loading-induced autophagy has been associated with survival and metabolism in various cell types.^154^ Cyclic changes in mechanical stimulus and its subsequent change in autophagy are indispensable to the network development of osteocytes.^155^

## Autophagy is involved in bone resorption

Bone resorption is conducted by multinucleated osteoclasts, which are derived from hematopoietic stem cells upon stimulation with macrophage colony-stimulating factor (M-CSF) and RANKL (receptor activator of nuclear factor kappa-B ligand; also known as TNFSF11, tumor necrosis factor ligand superfamily member 11).^114,156,157^ When functioning, osteoclasts polarize to form a ruffled border at the cell-bone interface. Numerous sealed-off compartments are formed under the ruffled border, across which degradative enzymes are secreted onto the bony surface. The degraded bony matrix is then transported into osteoclasts via endocytosis for recycling.^158^ As mentioned above, autophagy is actively involved in both the differentiation and functioning of osteoclasts.^132^

### Autophagy in the regulation of osteoclastogenesis

Osteoclasts originate from hematopoietic mononuclear myeloid stem cells, which in most cases reside in the marrow cavity. When the remodeling of the bony tissue is orchestrated and bone resorption is physiologically required or pathologically enforced, these mononuclear cells commit to a multinuclear osteoclastic phenotype and migrate onto the bony surfaces that are about to be remodeled.

The differentiation from adherent mononuclear cells into active osteoclasts is initiated by signal stimuli, including colony-stimulating factor 1 (CSF1) and TNFSF11/RANKL.^159^ At the initiation of osteoclastogenesis, mononuclear cells fuse with each other to become multinucleated giant cells.^160^ The recruitment of differentiating cells to the bone-remodeling site is dependent on chemokines, including CXCL12 (chemokine (C–X–C motif) ligand 12) and S1P (sphingosine 1-phosphate).

As mentioned above, autophagic activity upregulation protects cells from apoptosis. Under in vitro hypoxia and high oxidative stress conditions induced by glucocorticoids, the level of autophagic activity increased to reduce cell stress and thus protect osteoclast formation and survival.^161–163^ Similarly, under an in vivo environment, a regional hypoxic environment is expected, while differentiating osteoclasts migrate to the bone surface. Park et al.^164^ showed that hypoxia promoted increased expression of BNIP3, which consequently upregulated autophagic flux and MAP1LC3 recruitment to autophagosomes.^165^ Conversely, increased autophagic activity was correlated with enhanced osteoclast differentiation.^163^ These data suggested a role for the HIF1A-BNIP3 signaling pathway in mediating osteoclast differentiation.^162^

### Autophagy and osteoclast functioning

When the bone-resorption program is activated, the terminally differentiated osteoclasts tightly adhere to the bony surface, and such attachment is achieved through specialized structures formed on the contact side of the osteoclasts named podosomes. Functional proteins, including actin filaments, F-actin, and actin monomers, serve as critical anchors for osteoclast attachment. The actual resorption is accomplished by the generation and secretion of lysosomes containing acids and proteases. The lysosomes migrate to the ruffled border between osteoclasts and the bone surface, fuse with the cell membrane at the podosomes, and externalize the hydrochloric acid and proteases. The acids dissolve the mineral contents of the bone, and proteases, including matrix metalloproteinases, decompose the collagen matrix.^166^

When functioning, the active osteoclast needs to orchestrate the synthesis and externalization of acids and enzymes and the internalization and decomposition of the resolved matrix. In addition to their role in osteoclastogenesis, autophagy has been proven indispensable in the functioning of osteoclasts. When the exogenous autophagy inhibitor bafilomycin is added to the culture medium of osteoclasts, the resorptive activity is sharply decreased.^167^ The autophagy-related proteins ATG5, ATG7, ATG4B, and MAP1LC3 have all been suggested to play critical roles in the activation of resorption function. Both in vivo and in vitro data suggest that ATG5 and ATG7 promote osteoclastic functioning and guide lysosomes to target the actin ring. Specific knockdown of genes related to autophagosome formation (*Atg5*, *Atg7*, *Atg*4b, or *Lc3*) in mononuclear osteoclast progenitors in mice leads to defects in lysosomal trafficking and formation of resorptive brush borders in osteoclasts and, consequently, downregulates bone-resorption activity and increases bone volume.^132^ Notably, neither ATG5 nor ATG7 affects osteoclastogenesis. In the specific knockdown models, the number of nuclei within osteoclasts, the presence of secretory lysosomes or even the expression of actin ring proteins was unaffected. In addition, ATG4B modulation of MAPILC3A blocked both the resorptive activity and expression of CTSK (cathepsin K). When ATG5 was knocked down, significant downregulation in CDC42 activity and actin-ring disruption were observed. When MAPILC3A was knocked down, actin ring formation, resorption activity, and CTSK release were all inhibited.^168^ Consequently, it has been proposed that autophagy-related proteins can regulate bone-resorbing activity via the MAP1LC3-CDC42-dependent axis, which influences actin ring formation and ruffled border organization of osteoclasts.

In addition to the RANK/RANKL axis, inflammatory pathways are involved in the differentiation and function of osteoclasts. The presence of TNF-α activates NF-κb signaling and promotes osteoclastogenesis and bone resorption activities, and this mechanism is partially mediated through autophagy.^169^

As mentioned above, p62 is an important protein responsible for target sequestration for autophagosomes. In humans, the p62 mutation is observed in ~40%–50% of patients with Paget’s bone disease (PD), a condition involving increased bone resorption by osteoclasts, followed by excessive and disorganized bone formation by osteoblasts.^170^ This phenotype has also been recapitulated in mice, where a mutation in the gene *Sqstm1* encoding the p62 protein results in excessive osteoclastic activity and a phenotype similar to Paget’s disease.^171^

**Fig. 5:**
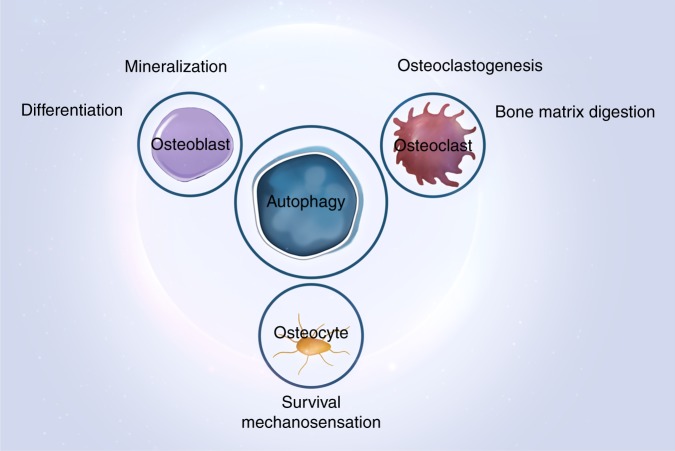
Autophagy and the functioning of three types of bone cells. Autophagy maintains the homeostasis of osteoblasts, osteoclasts, and osteocytes

**Fig. 6:**
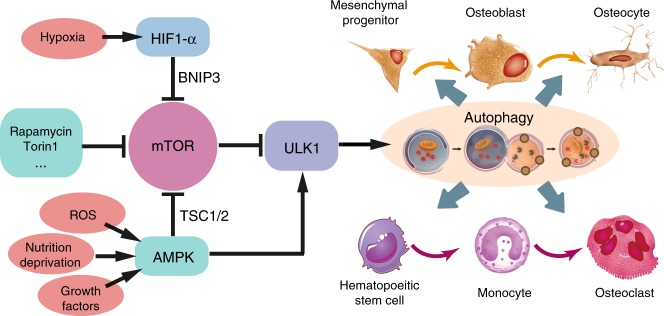
Signal pathways regulating the bone-related autophagic activity. mTOR and ULK1 serve as pivots in conducting stress signals and growth factors to downstream autophagy-related proteins

Taken together, these results indicate that fine-tuning the balance of autophagic activity in all three types of bone cells is pivotal in the maintenance of bone homeostasis (Figs 5, 6).^172^

## Autophagy in the onset of osteoporosis

In humans, bone mass accumulates during growth and reaches its peak in early adulthood. After that, both men and women start to lose bone mass due to a combination of intrinsic and extrinsic factors.^173^ When the bone mass drops to a level below the age-match average standard, a clinical diagnosis of osteoporosis is made. The incidence of osteoporosis-related fractures, associated with substantially increased morbidity and mortality and the costs of these fractures, has become a significant public health concern in many countries.^174–176^ As life expectancy increases globally, the number of osteoporotic fractures is expected to reach 6.3 million in 2050, compared with 1.7 million in 1990.^177^

Although the cause of osteoporosis is multifactorial, including genetic, hormonal, and nutritional factors, combined with people’s lifestyle choices, the basic biological mechanism of bone mass loss is the tilting of bone formation–resorption balance to the osteoclastic side.^178^ Bone tissue is under constant remodeling by the coordinated action of osteoclasts, osteoblasts, osteocytes, and bone-lining cells^13,179^ Any imbalance so that bone reabsorption exceeds formation can result in bone loss and subsequent osteoporosis.^180–183^

**Fig. 7:**
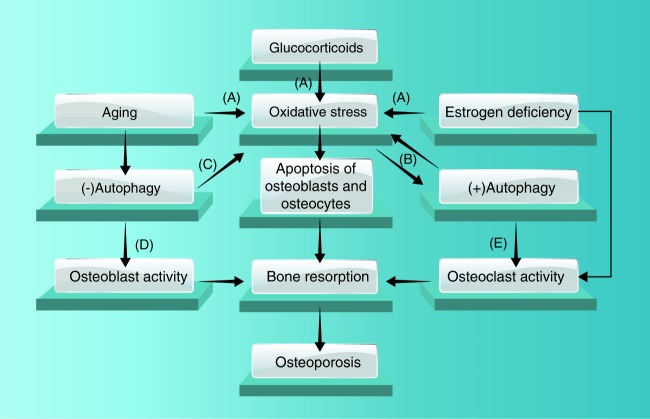
Pathological malfunction of autophagy triggers the onset of osteoporosis. Aging, estrogen deficiency, and glucocorticoids downregulate autophagic activity and thus lead to osteoporosis

Because autophagy is actively involved in the functioning of all bone cells, it is naturally correlated with multiple congenital and acquired bone abnormalities (Fig. 7). As mentioned above, disturbance in autophagy-related gene expression is associated with osteoclast malfunction and PD.^171^ Overactivation of autophagy has been associated with premature closure of craniofacial sutures.^184^ Thus, it is not surprising that autophagy plays a role in the onset and development of osteoporosis. In support of this hypothesis, a recent pathway-based analysis with genome-wide association study data suggested that variants in and around autophagy-related genes were associated with wrist bone mineral density.^162^

Aging is among the factors most closely associated with the onset of osteoporosis, which might be attributed to changes in hormones and oxidative stress with age.^185^ Correspondingly, the level of autophagic activity generally declines during aging,^186^ the observation of which is more notable in terminally differentiated cells, including osteocytes and osteoclasts.^187^ Thus, downregulated autophagic activity during aging might be one of the direct causal factors for the initiation and progression of age-related osteoporosis.^188^ This hypothesis has been supported in multiple animal models.

An important mechanism that is involved in age-related degeneration is the upregulation of systemic inflammation, which is now frequently termed inflamm-aging.^189^ Specific to bone, persistent activation of innate inflammatory pathways, for example NF-kB signaling, exerts diverse negative effects on bone mass maintenance, including inhibition of osteoblast differentiation and mineralization and abnormal activation of osteoclastic activities.^190,191^ Tumor necrosis factor-α (TNF-α) is a strong proinflammatory cytokine and activator of the NF-κB signaling pathway. The presence of TNF-α induces impeded osteoblast differentiation and enhanced osteoclastic differentiation and thus mediates phenotypes of bone loss in multiple scenarios.^190^ The negative effect of TNF-α on bone maintenance is at least partially mediated by autophagy.^192^ In a model of arthritis, TNF-α induced increased expression of *Atg7* and *Beclin-1*, which led to enhanced osteoclastogenesis and bone-resorption activities.^169^

Generally, autophagy-related proteins are involved in inflammatory signaling through two major mechanisms. One of the mechanisms is autophagy dependent, where ATG proteins eliminate the damaged mitochondria and thus reduce the intracellular level of ROS and RIG-I signaling. Another mechanism is independent of the autophagic process, and ATG proteins interact directly with molecules in inflammatory signaling. For example, the ATG5-ATg12 complex could bind to the caspase activation and recruitment domains of RNA-recognition molecules and subsequently downregulate the type I interferon (IFN) signaling induced by virus invasion.^193^ In another case, ATG proteins interact with cyclic GMP–AMP synthase and STING (stimulator of interferon genes) that mediate SNA-sensing immune signaling pathways. ULK1 could phosphorylate STING and inhibit STING-dependent cytokine formation and the inflammatory response.^194^ As a result, reagents that activate ULK1 are considered to have therapeutic potential for autoinflammatory disorders.^195^ Similarly, Beclin-1 suppresses interferon levels by binding to cyclic GMP–AMP synthase, and ATG9a antagonizes the formation of the STING–TBK1 complex and thus negatively regulates inflammatory signaling.^196,197^ At the same time, the activation of these inflammation-related RNA/DNA-sensing signaling pathways would turn on autophagic activities.^198^ As a result, ATG proteins both mediate intracellular inflammatory signaling and provide a feedback mechanism restraining excessive inflammatory responses.

In a rodent model of osteoporosis, the level of autophagy in osteoblasts was significantly downregulated,^199^ and a decreased level of autophagic activity in osteocytes has been associated with increased bone loss during aging.^200^ In a senile rat study, activation of autophagy in osteocytes and relief of the age-related osteoporosis state were achieved by systemic administration of rapamycin.^201^ In addition, as mentioned above, mice with osteocyte-specific deletion of *Atg7* demonstrated abnormally higher oxidative stress and increased bone loss at a young age, the phenotypes of which were similar to those caused by natural aging.^151^ The mTOR pathway effector ribosomal protein S6K1 (S6 kinase 1) serves as a negative feedback mechanism and curbs excessive autophagic activities. Mice with systemic deletion of S6K1 were resistant to aging-related decreases in bone density,^202^ and such resistance was evidently correlated with an amelioration in the decline of autophagic activity during aging.

When bone marrow mesenchyme cells were isolated for study, significantly lower levels of autophagic activity were observed in MSCs from aged animals compared with young animals. Accordingly, pharmacologically manipulating the autophagy level directly affects the differentiation capacity and direction of MSCs. For instance, treatment with the class III PI3K inhibitor 3-MA (3-methyladenine) downregulated the osteogenic and proliferative capacity of MSCs from young animals, while rapamycin treatment enhanced the capacity of aged MSCs.^25^

Estrogen deficiency is a common cause of osteoporosis, especially among postmenopausal women.^148^ In the estrogen-deficient rat model induced by ovariectomy, a significant inverse correlation was established between the autophagy level in osteocytes and the oxidative stress status and bone loss.^203^ Both monocyte-specific deletion of *Atg7* using *LysM-cre* mice and systemic delivery of the autophagy inhibitor chloroquine could effectively reduce the osteoclastic activity and mitigate bone loss among ovariectomized mice.^204^ In addition, osteoblast-specific suppression of autophagy in mice aggravated the bone loss associated with either aging or estrogen deprivation.

Glucocorticoid-induced osteoporosis is another of the most common forms of secondary osteoporosis. Oral glucocorticoid intake undermines the proliferative capacity of osteoblasts while enhancing the survival of osteoclasts and bone resorption and consequently results in low bone density and a higher risk of fracture. In a mouse model, glucocorticoid exposure reduced the number of autophagic osteoblasts by up to 75%.^205^ In addition, glucocorticoids might hinder intercellular communication among osteocytes via autophagy pathways. In addition, glucocorticoids promoted the increase in osteoclast number. Mice with osteoclast-specific deletion of autophagy-related proteins demonstrated resistance to glucocorticoid-induced bone loss.^204^

Moreover, multiple systemic metabolic dysregulation demonstrates osteoporotic phenotypes. Among patients with diabetes, impeded bone turnover is observed along with decreased bone density.^206^ Reduced autophagic activity in islets is characteristic of diabetes, and several autophagy activators demonstrated therapeutic potential in diabetes.^207^ Obesity also contributes to the prevalence and severity of diabetes and bone health. In multiple animal models, a high-fat diet and obesity favor adipogenic differentiation of MSCs and result in a decrease in bone volume.^208^ Defects in autophagy homeostasis are implicated in the onset of obesity.^209^

In general, the reduction of autophagy appears to promote increased oxidative stress, causing bone loss, and osteoporosis, whereas an increase in the autophagic pathway inhibits this effect.^151,200,201^

## Potential and obstacles in modulating autophagy as an osteoporosis therapy

Given all these evident correlations between the autophagic activities and functioning of bone cells, it is almost natural to anticipate the possibilities of therapeutics based on autophagy modulation. According to available basic and clinical studies, factors regulating autophagy have been widely associated with bone mass and strength changes induced by either aging, estrogen deficiency, or glucocorticoid treatment. The mechanisms underlying these correlations supply many potential therapeutic targets for rescuing osteoporosis.^210^ Active efforts have been made to develop therapeutic agents that modulate autophagy in the form of compounds or cell­permeable peptides.^211,212^ Preliminary positive outcomes have been reported in certain metabolic diseases. For example, upregulation of autophagy has shown some improvement in neurodegenerative disorders, in which reduced autophagy leads to accumulation of inclusion bodies.^213^ For osteoporosis, systemic adjustments of the autophagy level have demonstrated efficacy to varying degrees in multiple animal models.

Systemic delivery of autophagy-regulating drugs is the first strategy to be tested on animal models. In an age-related osteoporosis rodent model, systemic treatment with rapamycin demonstrated a protective effect on bone loss, at least in part. Such protection has been correlated with increased autophagy levels in osteocytes with upregulated LC3 turnover. When two-year-old rats were intraperitoneally delivered rapamycin, an autophagy inducer, for 12 weeks, both the ratio of apoptotic osteocytes and the number of osteoclasts decreased. Moreover, the autophagic activity in osteocytes was upregulated as demonstrated by an increase in the number of *Lc3*-positive osteocytes and LC3 turnover. Accordingly, a significant increase occurred in the mineral density and mineral apposition rate of the trabecular bone.^201^ Similarly, rapamycin exerts similar enhancement on bone formation during bone fracture repair, and again, the mechanism is related to increased autophagy and avoidance of bone cell apoptosis.^214^

In addition to aging, the glucocorticoid-induced osteoporosis model has been tested with autophagy modulators.^215,216^ Autophagic activity demonstrated a protective effect on MSCs against glucocorticoid-induced inhibition in osteogenic and proliferative potential. The autophagy inhibitor 3-MA suppresses proliferation and worsens apoptosis of MSCs, resulting in decreased bone mass. The potential mechanism underlying the effectiveness of rapamycin on glucocorticoid-induced osteoporosis has been further illuminated in vitro.^217^ Glucocorticoids target mTOR signaling and downregulate autophagy to modulate the proliferation and apoptosis of osteoblasts and osteocytes. Rapamycin also affects the mTOR and the subsequent autophagy pathways, indicating a potential competitive mechanism between glucocorticoids and rapamycin.

In addition to rapamycin, several other therapeutics exert effects via autophagy regulation, at least in part. Sclerostin antibody could effectively relieve the osteoporotic state induced by glucocorticoids, and such effects have been strongly correlated with upregulated levels of autophagy in osteoblasts.^205^ Systemic delivery of sclerostin antibody protected mice from glucocorticoid-induced osteoblast apoptosis and bone loss. Recently, NAD^+^ precursors, e.g., nicotinamide mononucleotide (NMN) and nicotinamide riboside (NR), have been proven to elevate autophagy-related gene expression by activating the NAD^+^ salvage pathway and de novo synthesis and are deemed promising therapeutic candidates to treat autophagy dysfunction.^218,219^ Thus, modulation of autophagy has potential for developing new pharmaceutical solutions for the treatment of glucocorticoid-induced osteoporosis.^220^

Parathyroid hormone (PTH) tightly regulates the homeostasis and functioning of all types of bone cells, and has been approved for clinical treatment of osteoporosis. The protective effect against bone loss from PTH has also been associated with autophagic activity and signaling. In vitro data showed that PTH enhances osteocyte survival following glucocorticoid treatment by upregulating the autophagic activity level.^19^ In a rat model, PTH relieved bone damage from osteoarthritis by enhancing autophagy in bone cells,^19^ and this effect, again, occurs via the mTOR pathway.^221^

Although these data support the therapeutic potential of autophagy, there are several obstacles that must be addressed before the clinical application of autophagy modulators in the prevention or treatment of osteoporosis.

First, the onset and progression of the osteoporosis state involve dysregulation of multiple osteogenic and osteoclastic pathways—Wnt signaling,^222^ TGF signaling,^112,115^ BMP signaling, and^223^ levels of various hormones,^224^ in addition to autophagy. Modulation of autophagic activity alone might not be sufficient to efficiently influence the overall remodeling configuration of the skeletal system.

Another point to be considered in the development of therapeutic autophagy modulators to treat patients with osteoporosis is that systemic alterations of autophagic activity might exert diverse, sometimes adverse, effects on different organs or metabolic processes. Consequently, systemic autophagy modulators could have unanticipated side effects on patients. One possible direction for future development of autophagy-based therapeutics is to develop safe, organ-specific autophagy modulators.

Furthermore, autophagy modulation has been reported to have various or even contradictory effects on different cells or in different environments over time.^225^ The most notable example is that the upregulation of autophagy enhances cellular functions in both osteoblasts^18^ and osteoclasts^162^ in vitro, promoting the secretion and resorption of the bony matrix, respectively. Such a cell-specific effect is also evident in vivo. Although most studies reported reduced autophagy with increased bone loss, it has recently been observed in mice that pharmacological or genetic inhibition of autophagy reduces osteoclastogenesis and bone resorption, inhibiting bone loss caused by ovariectomy or glucocorticoid treatment.^204^ Osteoclast-specific deletion of autophagy-related genes resulted in a phenotype immune to bone loss after treatment with either glucocorticoids or estrogen deficiency.^204^ The enhancement or debilitation of autophagic activities in patients with osteoporosis is still a major question under debate.

In addition, it has long been recognized that the level of autophagy must stay within a narrow range to maintain homeostasis.^156^ The exact range, however, could hardly be defined or measured. The balances between autophagy and other cellular activities are so delicate that either too high or too low a level of autophagy might threaten cell homeostasis and survival.^226^ The same signal might indicate different cell behaviors via autophagy. For example, glucocorticoids could increase the apoptosis ratio and suppress intercellular communication among osteocytes, while the former effect is achieved by downregulating autophagic activity. The latter effect is also autophagy dependent.^227^ Unrestrained induction or inhibition of autophagic flux might exacerbate the problem rather than resolve it.^228^ Thus, fine-tuning rather than broad-brush style interventions are preferred. In addition, autophagy modulators still have safety concerns that must be addressed before they can be adopted as therapeutics for patients. For example, rapamycin, the most studied autophagy stimulator, demonstrated a broad range of metabolic side effects when delivered systemically, including peripheral edema, hypertriglyceridemia, hypertension, and hypercholesterolemia.

Further complex issues include the variation among individuals in their response to autophagic modulation. For example, existing data suggest that autophagic modulation in the osteogenic cell lineage is gender- and age dependent at least to a certain degree.^229^ Analyses in mouse models revealed that although the level of autophagic activity in osteocytes decreases during aging in both males and females, the autophagy level in osteoblasts only decreases among females. In addition, while orchidectomy significantly downregulates the level of autophagic activity in osteoblasts, ovariectomy demonstrates no similar effect.

Finally, even in the same cell lineage, autophagy may be involved in contradictory cellular processes. For example, the negative effects of glucocorticoids on osteocyte activity are largely correlated with its suppression of overall autophagy level, while glucocorticoids rely on autophagic activity in degrading connexin 43 in osteocytes and blocking cell-to-cell communication. Thus, a simple on-and-off pattern might not be sufficient for ideal modulation of autophagy.

To further develop a clinically feasible modulatory strategy of autophagic activity to relieve osteoporosis, a better understanding of how autophagy regulates skeletal homeostasis at both the cellular and organismal levels and development of more sensitive and organ- and cell-specific therapeutic agents are needed.

## Conclusions

Autophagic catabolism modulates the survival and functioning of osteoblasts, osteocytes, and osteoclasts, and is thus critical to the maintenance of skeletal homeostasis. Aberrant autophagic activity leads to disruption of the bone-remodeling balance, which manifests as pathological states, including osteoporosis and osteopetrosis. Autophagy modulation has been shown to have therapeutic potential in the prevention and treatment of bone-related diseases. Nevertheless, future studies, especially large randomized, double-blind, placebo-controlled clinical trials, are needed to further confirm the possible relationship between autophagic dysfunction and osteoporosis in humans and to develop potential physiological and pharmaceutical therapies for bone diseases.
