# Correction to: Autophagy in bone homeostasis and the onset of osteoporosis

**DOI:** 10.1038/s41413-020-00114-0

**Published:** 2020-10-06

**Authors:** Xing Yin, Chenchen Zhou, Jingtao Li, Renkai Liu, Bing Shi, Quan Yuan, Shujuan Zou

**Affiliations:** State Key Laboratory of Oral Diseases, National Clinical Research Center for Oral Diseases, West China Hospital of Stomatology, Sichuan University, Chengdu, 610041 China

**Keywords:** Osteoporosis, Bone

Correction to: *Bone Research* 10.1038/s41413-019-0058-7, published online 3 October 2019

One important citation regarding Fig. 7 was missing in the original manuscript.^1^ Figure 7 was adapted from the publication by Florencio-Silva et al.^2^ The corrected legend is provided as below:

**Fig. 7** Pathological malfunction of autophagy triggers the onset of osteoporosis. Aging, estrogen deficiency, and glucocorticoids downregulate autophagic activity and thus lead to osteoporosis (Adapted from Florencio-Silva et al.^2^)
